# Dimethyl Sulfoxide (DMSO) Decreases Cell Proliferation and TNF-α, IFN-γ, and IL-2 Cytokines Production in Cultures of Peripheral Blood Lymphocytes

**DOI:** 10.3390/molecules22111789

**Published:** 2017-11-10

**Authors:** Lucas de Abreu Costa, Marcelo Henrique Fernandes Ottoni, Michaelle Geralda dos Santos, Agnes Batista Meireles, Valéria Gomes de Almeida, Wagner de Fátima Pereira, Bethânia Alves de Avelar-Freitas, Gustavo Eustáquio Alvim Brito-Melo

**Affiliations:** 1 Immunology Laboratory, Integrated Center for Health Research, Federal University of the Jequitinhonha and Mucuri Valleys (UFVJM), Diamantina, MG 39100-000, Brazil; lucas.farmac@gmail.com (L.d.A.C.); m.ottoni@yahoo.com.br (M.H.F.O.); michaellegsantos@gmail.com (M.G.d.S.); agnesbm@gmail.com (A.B.M.); valeria.g.almeida@gmail.com (V.G.d.A.); wagnerufvjm@gmail.com (W.d.F.P.); gbrito1998@yahoo.com (G.E.A.B.-M.); 2 Multicenter Graduate Program in Physiological Sciences/UFVJM Graduate Program in Pharmaceutical Sciences/UFVJM, Federal University of the Jequitinhonha and Mucuri Valleys, Diamantina, MG 39100-000, Brazil; 3 Institute of Science and Technology, Federal University of the Jequitinhonha and Mucuri Valleys, Diamantina, MG 39100-000, Brazil; 4 Graduate Program in Dentistry, School of Dentistry, Federal University of the Jequitinhonha and Mucuri Valleys, Diamantina, MG 39100-000, Brazil

**Keywords:** dimethyl sulfoxide, solvents, anti-inflammatory agents

## Abstract

Dimethylsulfoxide (DMSO) is an amphipathic molecule composed of a polar domain characterized by the sulfinyl and two nonpolar methyl groups, for this reason it is able to solubilize polar and nonpolar substances and transpose hydrophobic barriers. DMSO is widely used to solubilize drugs of therapeutic applications and studies indicated that 10% *v/v* concentration did not modify culture viability when used to treat human peripheral blood mononuclear cells (PBMC). However, some DMSO concentrations could influence lymphocyte activation and present anti-inflammatory effects. Therefore, the objective of this study was to evaluate the effect of DMSO on lymphocyte activation parameters. Cell viability analysis, proliferation, and cytokine production were performed on PBMC from six healthy subjects by flow cytometry. The results indicated that 2.5% *v/v* DMSO concentrations did not modify lymphocytes viability. DMSO at 1% and 2% *v/v* concentrations reduced the relative proliferation index of lymphocytes and at 5% and 10% *v/v* concentrations reduced the percentage of total lymphocytes, cluster of differentiation 4^+^ (CD4^+^) T lymphocytes and CD8^+^ T lymphocytes interferon-γ (IFN-γ), tumor necrosis factor-α (TNF-α) and interleukin-2 (IL-2) producers. Thus, it was concluded that DMSO has an in vitro anti-inflammatory effect by reducing lymphocyte activation demonstrated with proliferation reduction and the decrease of cytokine production.

## 1. Introduction

Dimethyl sulfoxide (DMSO, (CH_3_)_2_SO), presents a highly polar domain characterized by a sulfinyl group and two apolar methyl groups. Such characteristics provide amphipathic properties to the molecule [1]. DMSO is able to solubilize polar and nonpolar substances and transpose hydrophobic barriers, such as the plasma membrane. These properties are important for vehicle pharmacological compounds that act intracellularly [2]. Thus, DMSO is used as a solvent for pharmacological substances, as well as to several other applications such as therapeutic applications, excipient for veterinary therapeutic formulations, as control group for testing natural products, to treat cultured cells and in other experimental in vitro studies. Despite of the multiple applications of DMSO, its physiological and pharmacological effects are not fully understood [3].

DMSO is considered as a low toxicity solvent. According to Kloverpris et al. [4] DMSO can be used up to 10% *v/v* for one hour treatment of human peripheral blood mononuclear cells (PBMC) cultures without changing culture viability. However, some concentrations of DMSO could modify cell activation in culture. Cultures of cells extracted from rat liver treated with DMSO at 25% *v/v* increased the activity of lysosomal enzymes [5]. Human peripheral blood lymphocytes treated in vitro with 40% *v/v* DMSO increased the expression of selectins and the phosphorylation of proteins involved in intracellular activation pathways [6]. C-28/I2 human chondrocyte cultures treated with different DMSO concentrations for 12 h, indicated that concentrations higher than 1% *v/v* reduced the expression of interleukins 6 and 8 (IL-6 and IL-8) [7]. On intestinal cells (Caco-2), in vitro stimulation with an inflammatory cocktail and treatment with low concentrations of DMSO (0.1 to 0.5% *v/v*) reduced the expression of IL-6 and IL-1β inflammatory cytokines [3]. Such outcomes can indicate that different cell types respond very differently to DMSO concentrations and stimuli conditions. Another reported application for DMSO is cell cryopreservation [8], although in this case cell viability could be affected by the solvent proportion. Long-term cryopreservation of fibroblasts with 1% *v/v* DMSO reduced cell viability to 73%, while using 0.5% *v/v* DMSO, the fibroblasts viability was greater than 80% [9]. In another study, the treatment of immortalized mice macrophages (v-myc) with 2% *v/v* DMSO for 48 h increased cell death by apoptosis [10]. In cultures of different *Candida* species, 1% *v/v* DMSO did not modify cell viability, however the expression of cell wall proteins and the catalase activity were affected [11].

Despite its well-documented capacity to dissolve a wide range of chemical compounds, further investigations regarding DMSO’s pharmacological activities and reliable safe use concentrations are required. For this purpose and regarding previous studies that used DMSO doses ranging from 0.5 to 40% *v/v*, the main objective of this study was to verify DMSO concentrations that are not toxic and do not modify the activation of peripheral blood cells on in vitro cultures.

## 2. Results

### 2.1. Cytotoxicity Tests (Assays)

**Figure 1:**
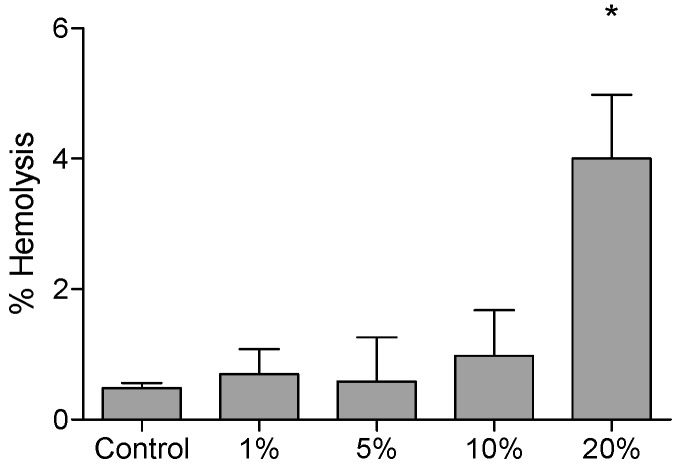
Hemolytic effect of different dimethyl sulfoxide (DMSO) concentrations (1, 5, 10, and 20% *v/v*). Hemolysis percentage after 4 h of incubation (*n* = 6 subjects). Results were expressed as mean and standard deviation (SD). * Statistically significant difference when compared to the control culture (*p* < 0.05, one-way ANOVA followed by Tukey’s post hoc test).

Hemolysis and trypan blue exclusion assays were performed to evaluate the cytotoxic effect of different DMSO concentrations. The results indicated that 20% *v/v* DMSO was hemolytic, presenting higher percentage value (4.0 ± 0.97%) when compared to spontaneous hemolysis in control group (0.48 ± 0.08%) (Figure 1).

**Figure 2:**
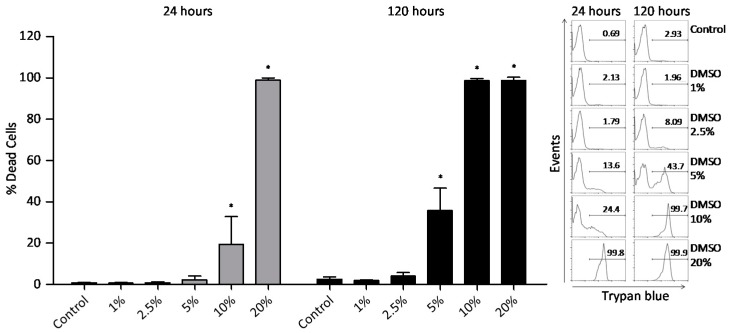
DMSO cytotoxic effect on peripheral blood mononuclear cells (PBMC) cultures at 24 and 120 h. Non-viable PBMC percentage (*n* = 6 subjects) in cell cultures not treated with DMSO (control) or treated with it at concentrations of 1, 2.5, 5, 10, and 20% *v/v*. Results from trypan blue exclusion test by flow cytometry technique were expressed as mean and SD. * Statistically significant difference when compared to the control culture (*p* < 0.05, One-way ANOVA followed by Tukey’s post hoc test).

To analyze DMSO cytotoxicity on PBMC, the trypan blue exclusion assay by flow cytometry was performed at 24 and 120 h. In the first 24 h, DMSO at 10% *v/v* increased PBMC death, while DMSO at 5% *v/v* increased PBMC death after 120 h (Figure 2).

### 2.2. Dimethylsulfoxide Effect on Lymphocytes Proliferation

**Figure 3:**
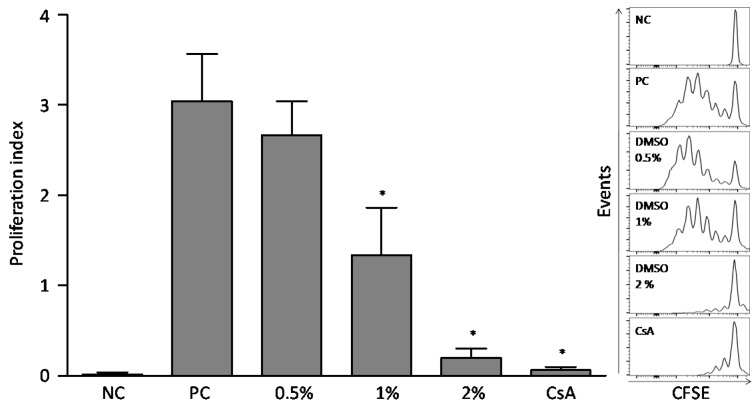
DMSO effect on lymphocyte proliferation using the 5-(and-6)-carboxy-fluorescein-succinimidyl ester (CFSE) label decay technique. NC = Negative Control not stimulated with PHA. PC = phytohemagglutinin (PHA)-stimulated control, CsA = inhibition control stimulated with PHA and treated with 5 μg/mL cyclosporine (CsA). The other cultures were stimulated with PHA and treated with different concentrations of DMSO (*n* = 6 subjects). Results were expressed as mean and SD. * Statistically significant difference when compared to the Positive Control (PC) culture (*p* < 0.05, one-way ANOVA followed by Tukey’s post hoc test).

After five days of culture (120 h), DMSO concentrations of 1% and 2% *v/v* reduced the lymphocyte proliferation index compared to the phytohemagglutinin (PHA)-stimulated positive control culture, in 55% and 90%, respectively. That reduction was also observed on the inhibition control culture, treated with cyclosporine (CsA). 0.5% *v/v* DMSO did not change the relative proliferation index of lymphocytes when compared to the control culture (Figure 3).

### 2.3. Effect of Dimethyl sulfoxide on the Production of Cytokines by Lymphocytes

**Figure 4:**
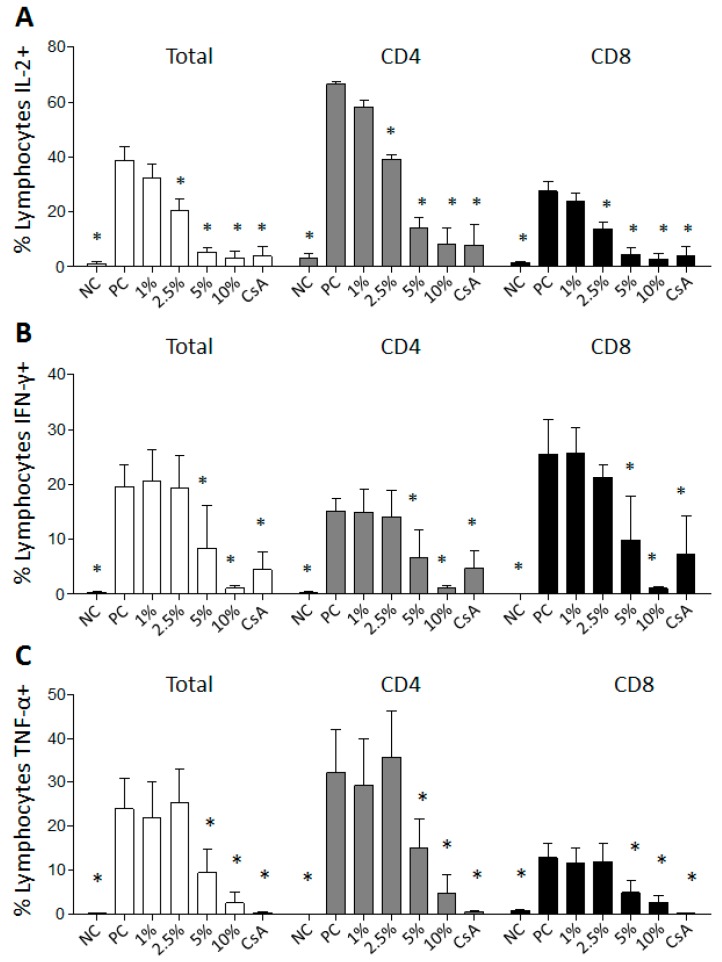
DMSO effect on the production of interleukin-2 (IL-2) (**A**), interferon-γ (IFN-γ) (**B**) and tumor necrosis factor-α (TNF-α) (**C**) in lymphocytes populations. Analysis of total lymphocytes, CD4^+^ and CD8^+^ relative percentage that were positives for cytokines in non-stimulated and untreated (NC), stimulated with phorbol-12-myristate-13-acetate (PMA) (PC) and stimulated with PMA cells culture in the last 4 h in a total of 8 h of DMSO treatment at 1, 2.5, 5, or 10% *v/v* or with 5 μg/mL of CsA. Results were expressed as mean and SD. * Statistically significant difference when compared to PC (*p* < 0.05, one-way ANOVA followed by Tukey’s post hoc test).

DMSO at 10 and 5% (*v/v*) concentrations decreased interleukin-2 (IL-2), tumor necrosis factor-α (TNF-α) and interferon-γ (IFN-γ) cytokines production in the total lymphocyte population and in cluster of differentiation 4^+^ (CD4^+^) and CD8^+^ T lymphocyte subsets (Figure 4). Furthermore, at the concentration of 2.5% (*v/v*), DMSO reduced IL-2 cytokine production by total lymphocytes and by CD4^+^ and CD8^+^ T lymphocytes subsets. The observed decrease of IL-2 cytokine for total lymphocytes and for CD4^+^ and CD8^+^ T lymphocytes subsets in stimulated cultures with phorbol-12-myristate-13-acetate (PMA) and treated with 2.5% (*v/v*) DMSO was 38, 40, and 50%, respectively (Supplementary Figure S1). For the IFN-γ production, the results revealed a decrease of 56% for total lymphocytes, 56% for CD4^+^ lymphocytes and 61% for CD8^+^ lymphocytes, when the cultures were treated with 5% DMSO *v/v* comparing to the positive control culture (Supplementary Figure S2). Similarly, 5% *v/v* DMSO reduced TNF-α production by the stimulated culture in 60% for total lymphocytes, 53% for CD4^+^ lymphocytes and 61% for CD8^+^ lymphocytes (Supplementary Figure S3).

## 3. Discussion

According to the results, DMSO presented toxicity to red blood cells at 20% *v/v* after four hours incubation. In the literature, the hemolytic concentration of DMSO is controversial, varying largely according to the experimental conditions adopted. Yi et al. [12] showed that small concentrations, as 0.2%, were able to induce hemolysis, however, Ansel and Leake [13] discussed the significant influence of the extracellular material on the hemolytic activity of DMSO. In that study, DMSO in aqueous solution caused hemolysis in rabbit erythrocytes at concentrations smaller than 2%, but no considerable hemolytic activity was observed when the cells were exposed to 25% DMSO in the presence of 0.6% saline. Therefore, the differences between the hemolytic concentration of DMSO established by Yi et al. (2017) [12] and the present study can be partially explained by differences in the extracellular material used.

The results showed that DMSO was toxic to PBMC at 10% *v/v* for the first 24 h and at 5% *v/v* after 120 h. Kloverpris et al. [4], suggested that the exposure time to DMSO can be more harmful than concentration itself, since it was observed that PBMC treated with 10% DMSO for 1 h did not affect cells viability, on contrast 0.2% DMSO increased cell death after seven days. The suggested mechanism for DMSO cytotoxicity is the effect on the physical properties of the phospholipids in membranes. As an amphipathic solvent, DMSO can interact with the plasma membrane allowing pores formation, which contribute to decrease membrane selectivity and increases cell permeability [14]. Moreover, our results demonstrated that the effect of DMSO treatment is not cell-specific because the DMSO concentrations values able to inhibit cytokine production were similar for total lymphocytes as for CD4^+^ and CD8^+^ T lymphocyte subsets. These results are in agreement to the study performed by Kloverpris et al. [4], where they demonstrated that long-term exposition to DMSO abolished similarly both CD4^+^ and CD8^+^ T-lymphocyte antigen-specific responses. Nevertheless, our data do not allow direct comparison of the DMSO effect on red and white blood cells due to the methodological differences used which include reagents and incubation times.

After antigenic activation, resting T lymphocytes undergo a process of clonal expansion (proliferation) and differentiate into effector cells. Proliferation precedes the effector responses of lymphocytes that are importantly orchestrated by cytokines [15]. Thus, the effect of different concentrations of DMSO on lymphocytes proliferation was evaluated, and the results showed that, at 1% *v/v*, DMSO was able to reduce the proliferation of PHA activated lymphocytes after five days of incubation. Kloverpris et al. [4] reported that DMSO at 1% and 1.5% concentration reduced by 50% the CD4^+^ and CD8^+^ T lymphocytes proliferative capacity when evaluated after seven days with Staphylococcal Enterotoxin type B (SEB) stimuli. Then, considering the DSMO antiproliferative effect, it is recommended a careful use for both in vitro and in vivo models.

IL-2 is synthesized by activated lymphocytes and acts as an autocrine growth factor in cells promoting clonal expansion and proliferation [16,17,18]. Since DMSO reduced lymphocyte proliferation, we investigated the effect of different concentrations on IL-2 cytokine production. The results demonstrated the reduction of total lymphocytes-IL-2^+^, as well as CD4^+^IL-2^+^ and CD8^+^IL-2^+^ T lymphocytes in cultures stimulated with PMA and treated with 2.5% *v/v* DMSO. Part of the DMSO anti-proliferative effect could be attributed to its action over the production of IL-2 cytokine. Santos et al. (2015) [19] observed that DMSO up to 0.5% *v/v* did not modify IL-2 production in PBMC cultures after stimulation with PHA for 36 h. However, its effect observed on cell proliferation cannot be attributed only to the reduction of IL-2 cytokine production, since other many mediators, such as Ca^2+^ and NF-κB, are also involved in cell proliferation stimulation [20,21,22].

Cytokines are important mediators responsible for the establishment, maintenance, and resolution of inflammatory reactions [23] and were further investigated concerning the effect of DMSO treatment on cultures stimulated with PMA. PBMC incubated for eight hours at different DMSO concentrations received PMA stimulus in the last 4 h, and then the production profile of proinflammatory cytokines IFN-γ and TNF-α were evaluated. We observed that 5% *v/v* DMSO substantially reduced all evaluated cytokines of total lymphocytes and CD4^+^ and CD8^+^ T lymphocytes subsets. These results can be attributed to DMSO since the cell viability was not affected by the treatment of DMSO at 5% *v/v* (Figure 2). Although the treatment with 10% DMSO for 8 h reduced the production of proinflammatory cytokines, we cannot exclude the possibility of cell death because the PBMC viability data demonstrated that the 10% DMSO treatment, for 24 h, induced death of approximately 20% of the cells. Another consideration is that the viability experiments were conducted with no stimulation while cytokine analyses were performed under PMA stimulation, a mitogen that commonly modifies cell morphology and could induce a low rate of cell death by itself. The simultaneous use of cell viability dyes in the experiment could clarify this issue. Specially, at the 10% *v/v* concentration, the inhibitory effect on IFN-γ production was nearly 5 times greater than the cyclosporine effect for the assessed cell populations. Avelar-Freitas et al. [24] observed that concentrations up to 1% *v/v* of DMSO did not change IFN-γ and TNF-α production when they were evaluated in the cytoplasm of human lymphocytes and neutrophils stimulated with PMA for 4 h. When evaluating DMSO anti-inflammatory activity over whole blood samples using ELISA assay, during 7 h incubation, Elisia et al. (2016) [25] verified that DMSO reduced the secretion of 13 cytokines important in inflammatory response. It was shown that 0.5% DMSO concentration is able of inhibit IFN-γ secretion, while 2% inhibits TNF-α secretion. In a different way, it was evaluated in the present study that IFN-γ and TNF-α secretion were inhibited by 5% DMSO specifically in total lymphocytes, CD4^+^ and CD8^+^ T lymphocytes subsets. An important aspect about DMSO and the suppression of proinflammatory cytokines is that the solvent could enable the differentiation and activation of the effector function of regulatory T cells (Treg: CD4^+^ CD25^+^ Foxp3^+^), that are relevant cells in the regulation of proinflamatory cytokine production. The immunosuppressive activity of DMSO was demonstrated by Lin et al. [26] using in vivo and in vitro models. They determined that solvent treatment decreases the percentage of IFN-γ-producing T lymphocytes and increases the percentage of Treg cells. In addition, Huang et al. [27] reported that different concentrations of DMSO stimulated the production of the immunosuppressive cytokine transforming growth factor-β (TGF-β) as well as its receptors. In the present study, we demonstrated the effect of DMSO on reducing proinflammatory cytokines production by lymphocytes, as well as reducing proliferation, highlighting a possible anti-inflammatory effect for DMSO.

## 4. Materials and Methods

### 4.1. Study Subjects and Biological Samples

Approximately 10 mL of venous blood sample from healthy donors (*n* = 6 subjects) was drawn by venipuncture into heparinized tubes following these blood-drawing criteria: no reported infection or symptoms of infection for seven days prior to the sample collection, subject reported adequate sleep (6–9 h), no exercise or alcohol use for 24 h prior to withdrawal of the blood sample, no topical corticosteroid or aspirin use for the previous 48 h, no systemic antihistamines or corticosteroid use for one week prior to obtaining the sample and no immunizations during the previous three weeks. All subjects included filled the informed consent, and the local Ethics Committee approved the realization of the present study (CEP/UFVJM-24530913.6.00005108).

### 4.2. Hemolysis Tests

To evaluate the toxicity of different DMSO concentrations to human red blood cells, the hemolysis test was performed as described. Total blood samples were diluted (1:20), adding 500 μL of total blood to 10 mL of phosphate buffered saline (PBS) in a beaker and the suspension was gently stirred on a magnetic stirrer throughout the course of the test. Then, DMSO at final concentrations of 1, 2, 5, 10, and 20% *v/v* were incubated with 100 μL of red blood cell suspension. Ultrapure water was added to cultures containing the cell suspension and DMSO, with the same final concentrations to provide the positive control that represents 100% hemolysis. Controls (blank) were made with DMSO at the tested concentrations. Spontaneous hemolysis cultures (negative control) consisting of PBS and 100 μL of red blood cell suspension with their respective blanks (PBS only) were also performed. Cultures were incubated in polystyrene tubes for 4 h at 37 °C and 5% CO_2_. After that time, the tubes were centrifuged (7 min; 500 g; 22 °C) and 200 μL of the supernatant was transferred to a flat bottom 96-well plate and absorbance was measured at 540 nm. The result of the hemolysis test was a hemolysis percentage obtained by the equation:

$$\%\;\mathrm{Hemolysis}=\frac{\left(Absorbance\;test-absorbance\;control\;negative\right)\times 100}{\left(\mathrm{Absorbance}\;\mathrm{of}\;\mathrm{the}\;\mathrm{positive}\;\mathrm{control}\;-\;\mathrm{Absorbance}\;\mathrm{of}\;\mathrm{the}\;\mathrm{negative}\;\mathrm{control}\right)}$$

### 4.3. Peripheral Blood Mononuclear Cells (PBMC) Isolation

PBMC were isolated by centrifugation using Ficoll-Histopaque-1077 (Sigma-Aldrich Corporation, St. Louis, MO, USA) as described by Bicalho et al., (1981) [28] and adapted as follows. Briefly, the blood was gently added over Ficoll-Paque and centrifuged at 520 *g*. PBMC were collected after Ficoll separation and washed three times with PBS (0.015 M, pH 7.4) and cell suspension was adjusted to 1.0 × 10^7^ cells/mL.

### 4.4. Peripheral Blood Mononuclear Cells (PBMC) Viability Analysis

**Figure 5:**
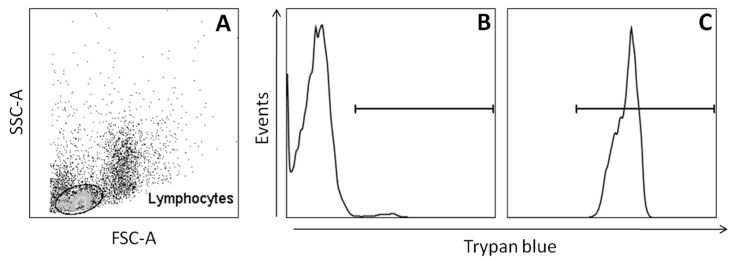
Computational strategy used to evaluate the percentage of dead cells by flow cytometry using trypan blue. Lymphocytes gating (**A**); selection of lymphocytes-trypan blue^+^ in a culture with low percentage of dead cells (**B**); and high percentage of dead cells (**C**).

PBMC (5 × 10^5^) were cultured in Roswell Park Memorial Institute-1640 (RPMI-1640) medium (Sigma-Aldrich Corporation, St. Louis, MO, USA) supplemented with 2 mM l-glutamine, 10% fetal calf serum (FCS) (Gibco by Thermo Fisher Scientific, Waltham, MA, USA) and antibiotic–antimycotic cocktail (penicillin G 100 IU/mL, streptomycin 100 μg/mL and amphotericin B 250 ng/mL) (Sigma-Aldrich Corporation). Cells were treated with DMSO at concentrations of 1, 2.5, 5, 10, and 20% *v/v* (Sigma-Aldrich Corporation) in a humidified incubator (Ultrasafe, Biosystems, Curitiba, Paraná, Brazil) at 37 °C with 5% CO_2_ air atmosphere for 24 h or 120 h. The cell culture control was composed by untreated PBMC. PBMC were washed with PBS (200 g, 7 min, 4 °C) and re-suspended in 0.5 mL PBS. Then, 10 μL of the cell suspension was mixed with 190 μL of 0.002% trypan blue (Sigma-Aldrich Corporation) and analyzed by flow cytometry (FACScan, Becton Dickinson BD Biosciences, San Jose, CA, USA) [29]. 10,000 events were acquired in the region corresponding to lymphocytes. CellQuest™ software (version 2.0, Becton Dickinson BD Biosciences, San Jose, CA, USA) was used for data collection and analyses (Figure 5). Cell viability was calculated by dividing the number of viable cells by the total cell number.

### 4.5. Proliferation Analysis with 5-(and-6)-Carboxy-fluorescein succinimidyl ester (CFSE) Label Decay Technique

The effect of DMSO on the proliferative lymphocyte response to PHA stimulation was evaluated by 5-(and-6)-carboxyfluoresceinsuccinimidyl ester (CFSE) fluorescence decay assay [30,31]. PBMC (1 × 10^7^) were re-suspended in phosphate buffer saline/bovine serum albumin (PBS/BSA) 0.1% and labeled with 10 μM CFSE (Sigma-Aldrich Corporation) for 10 min at 37 °C. CFSE-stained PBMC (5 × 10^5^; *n* = 6 subjects) were cultured in RPMI-1640 containing 10% FCS (Gibco, by Thermo Fisher Scientific), 2 mM l-glutamine, antibiotic-antimycotic cocktail (Sigma-Aldrich Corporation), either with or without phytohemagglutinin (PHA) (1 μg/mL). Cells also were stimulated with PHA in combination with different DMSO concentrations (0.5–1% and 2% *v/v*). Untreated PBMC were used as the non-stimulated cell culture control (NC). The cells were kept in a humidified incubator with a 5% CO_2_ air atmosphere for five days at 37 °C. Samples were analyzed on a BD FACSCanto™ II (Becton Dickinson BD Biosciences, San Jose, CA, USA) with 50,000 events acquired. Data acquisition and analysis were performed using software BD FACSDiva (v6, Becton Dickinson, BD Biosciences) and FlowJo software (v10.0.7, FlowJo LLC, Ashland, OR, USA), respectively. The proliferative index was then calculated from CFSE fluorescence histograms using the following formula [32]:

Proliferative index = 100−YY,

being *Y* (%) = *X0* + *X1/2* + *X2/4* + *X3/8* + *X4/16* + *X5/32* + *X6/64*; *X0* represents the percentage of T cells that did not divide and *X1–6* represents the maximum gradual division.

### 4.6. Analysis of the Cytokines Production by Lymphocytes

**Figure 6:**
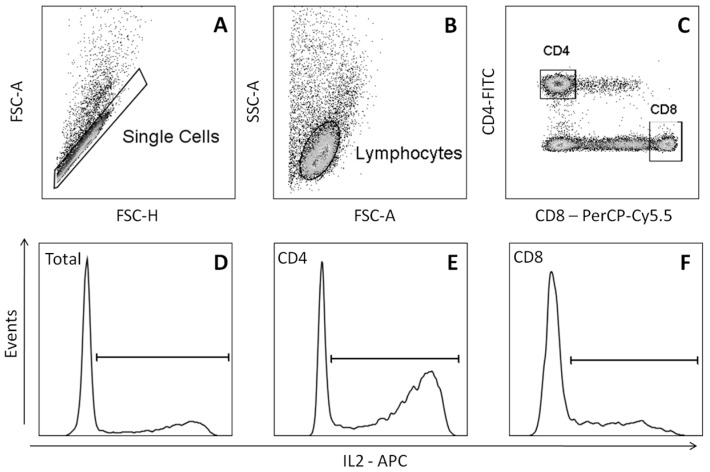
Computational strategy used to evaluate the percentage of positivity of cytokines on lymphocytes. Dot plot graphs were used sequentially for the selection of single cells (**A**); total lymphocytes (**B**); and CD4^+^ or CD8^+^ lymphocytes (**C**). In the sequence, histograms were used in the analysis of the percentage of cytokine-stained total lymphocytes (**D**), CD4^+^ lymphocytes (**E**) and CD8^+^ lymphocytes (**F**).

PBMC (5 × 10^5^) (*n* = 6 subjects) were cultured in RPMI-1640 containing 10% FCS (Gibco, by Thermo Fisher Scientific), 2 mM l-glutamine and antibiotic-antimycotic cocktail (Sigma-Aldrich Corporation). The cells were pretreated for four hours with PBS (negative control) or DMSO (1%, 2.5–5% and 10% *v/v*). In the next four hours, it was added 25 ng/mL phorbol-12-myristate-13-acetate-PMA (Sigma-Aldrich Corporation), 1 ng/mL ionomycin (Sigma-Aldrich Corporation) and 1 μg/mL Brefeldin-A (Sigma-Aldrich Corporation) to the stimulated cultures. Untreated PBMC were used as the non-stimulated cell culture control (NC). The cells were maintained at 37 °C in a humidified incubator (Ultrasafe, Biosystems) with a 5% CO_2_ atmosphere for 8 h. Cells were then fixed and incubated with anti-CD4 surface human antibodies labeled with fluorescein isothiocyanate (FITC) (BD PharMingen, San Diego, CA, USA) and anti-CD8 surface human antibodies labeled with PerCP-Cy5.5 (BD PharMingen, San Diego, CA, USA). After that, PBMC were permeabilized and incubated with monoclonal antibodies (mAb) conjugated with specific fluorochromes for IFN-γ (mAb-IFN-γ-PE-Cy7), TNF-α (mAb-TNF-α-PE), and IL-2 (mAb-IL10-APC,) cytokines (all from BD PharMingen). The expression of cytokines by lymphocytes was evaluated using a BD FACSCanto™ II (Becton Dickinson BD Biosciences) with 30,000 events recorded (Figure 6). Data acquisition and analysis were performed using software BD FACSDiva (v6, Becton Dickinson, BD Biosciences) and FlowJo software (v10.0.7, FlowJo LLC, Ashland, OR, USA), respectively.

### 4.7. Statistical Analyses

GraphPad Prism, version 5.0 for Windows (GraphPad Software, La Jolla, CA, USA) was used for statistical analysis. One-way ANOVA with Tukey’s post hoc test were performed. *p* values lower than 0.05 were considered to be statistically significant. Data was reported as means and standard deviation.

## Supplementary Materials

Supplementary materials are available online.
