# Supplementary material for: Dimethyl Sulfoxide (DMSO) Decreases Cell Proliferation and TNF-α, IFN-γ, and IL-2 Cytokines Production in Cultures of Peripheral Blood Lymphocytes
Source: Molecules. 2017 Nov 10;22(11):1789. doi: 10.3390/molecules22111789 (PMC6150313; doi:10.3390/molecules22111789)
Supplement: Supplementary file 1 [file molecules-22-01789-s001.pdf]

molecules

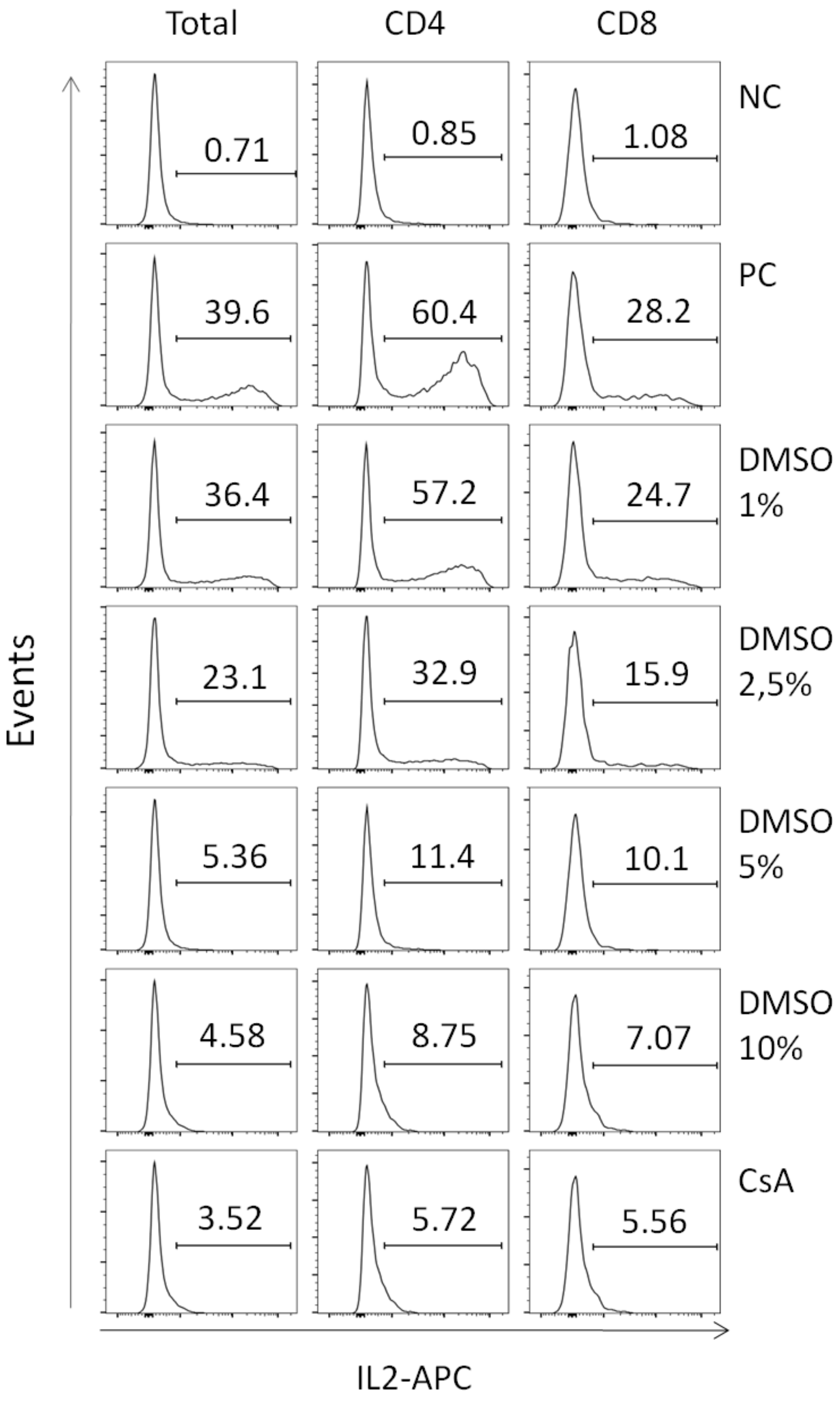

Supplementary figure 1. Representative histograms of the percentage of lymphocytes-IL-2+ by flow cytometry in untreated cultures (NC), stimulated with PMA (PC), stimulated with PMA in the last 4 hours in a total of 8 hours of DMSO treatment at 1; 2.5; 5. or 10% v/v or 5 μg/mL of cyclosporine (CsA).

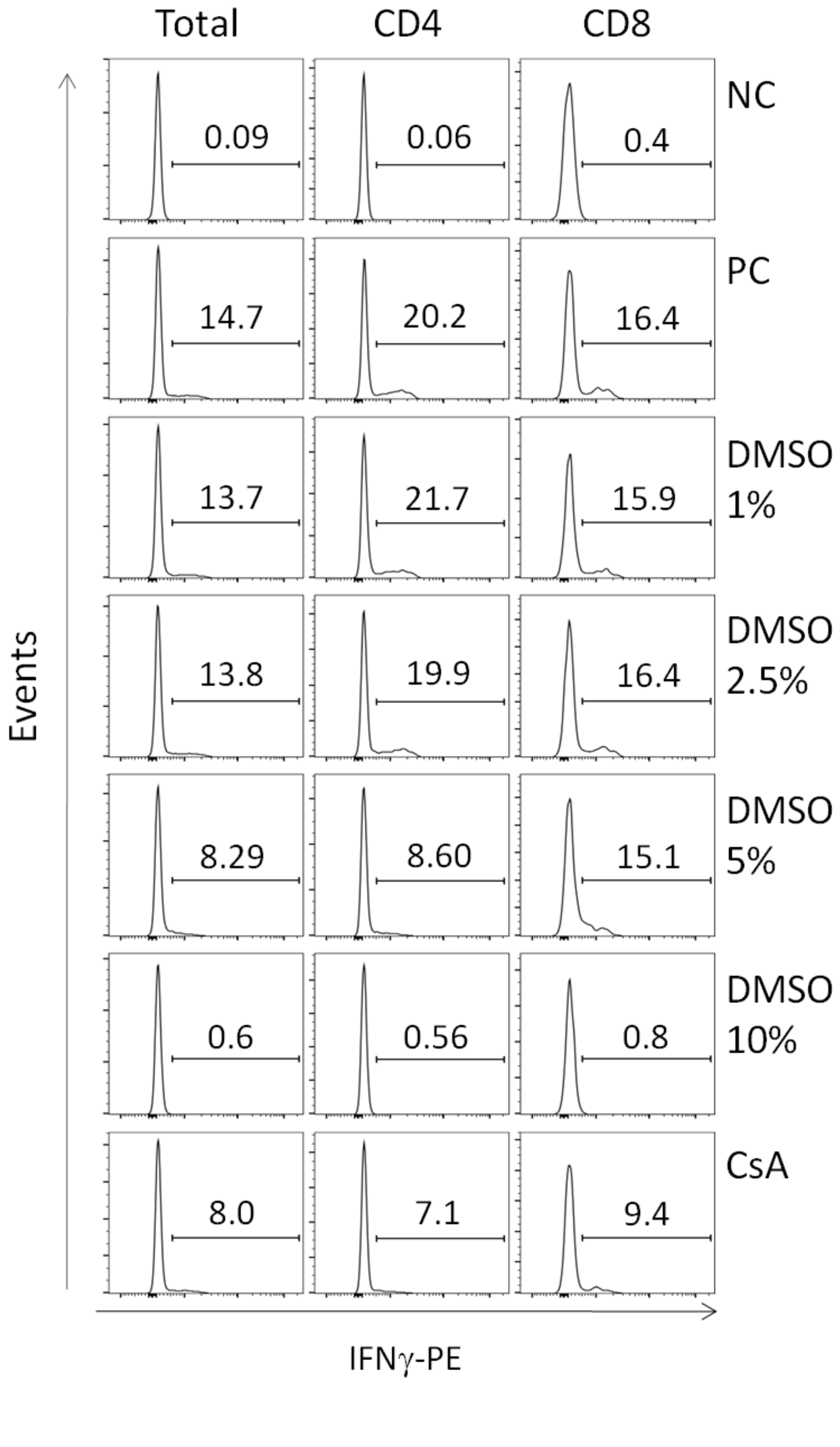

Supplementary figure 2. Representative histograms of the percentage of lymphocytes-IFN-γ by flow cytometry in untreated cultures (NC), stimulated with PMA (PC), stimulated with PMA in the last 4 hours in a total of 8 hours of DMSO treatment at 1; 2.5; 5. or 10% v/v or 5 µg/mL of cyclosporine (CsA).

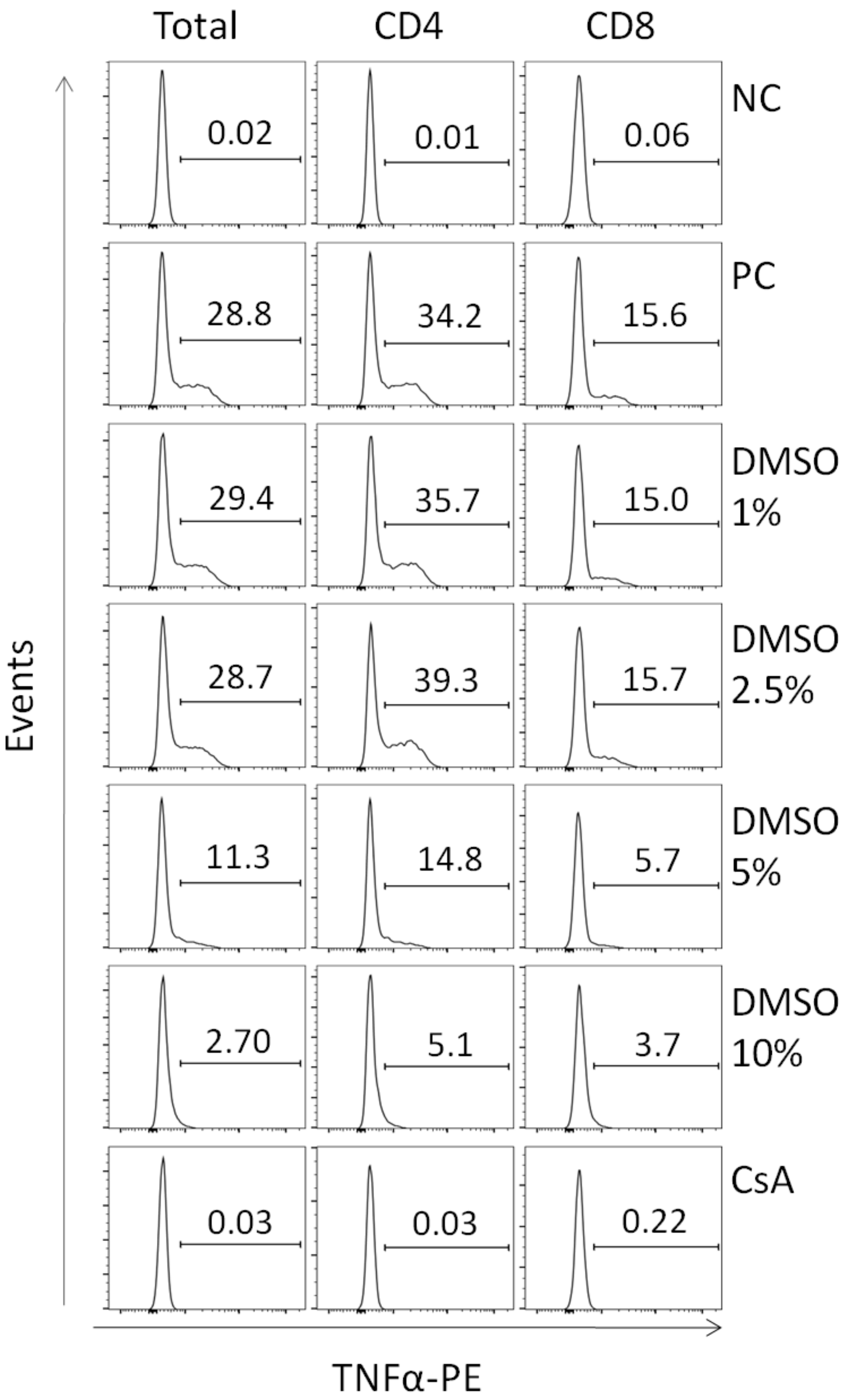

Supplementary figure 3. Representative histograms of the percentage of lymphocytes-TNF-α^{+} by flow cytometry in untreated cultures (NC), stimulated with PMA (PC), stimulated with PMA in the last 4 hours in a total of 8 hours of DMSO treatment at 1; 2.5; 5. or 10% v/v or 5 μg/mL of cyclosporine (CsA).
